# Level of adherence to ocular hypotensive agents and its determinant factors among glaucoma patients in Menelik II Referral Hospital, Ethiopia

**DOI:** 10.1186/s12886-016-0316-z

**Published:** 2016-08-02

**Authors:** Tesfay Mehari, Abeba T. Giorgis, Workineh Shibeshi

**Affiliations:** 1 Department of Pharmacy, College of Health Sciences, Mekelle University, Mekelle, Ethiopia; 2 Department of Ophthalmology, School of Medicine, College of Health Sciences, Addis Ababa University, Addis Ababa, Ethiopia; 3 Department of Pharmacology and Clinical Pharmacy, School of Pharmacy, College of Health Sciences, Addis Ababa University, Addis Ababa, Ethiopia

**Keywords:** Adherence, Ocular hypotensive agents, Glaucoma

## Abstract

**Background:**

Good adherence to ocular hypotensive agents is important to control intraocular pressure and hence to prevent progressive glaucomatous optic nerve head damage. Periodic investigation of adherence is crucial in glaucoma treatment. The purpose of this study was to assess level of adherence to ocular hypotensive agents and to identify factors affecting adherence among glaucoma patients at a tertiary public eye care center.

**Methods:**

The study was a hospital-based cross-sectional study that was conducted in Menelik II Referral Hospital from June 1, 2015 to July 31, 2015. A systematic random sampling technique was used to select 359 study participants from the source population. The study patients were interviewed and their medical charts were reviewed using a pretested structured questionnaire. Adherence was assessed using Morisky Medication Adherence Scale - 8 and adherence determinant factors were identified using multivariate binary logistic regression analysis. The association was declared statistically significant at *p* < 0.05.

**Results:**

Among the 359 study glaucoma patients, 42.6 % were adherent to their prescribed hypotensive agents. Higher educational level (AOR = 4.60, 95 % CI: 1.01–21.03, *p* < 0.049), being self - employed (AOR = 6.14, 95 % CI: 1.37–27.50, *p* < 0.018) and taking lesser frequency of drops (AOR = 2.89, 95 % CI: 1.25–6.66, *p* < 0.013) were significantly associated with adherence, whereas being a farmer (AOR = 0.07, 95 % CI: 0.01–0.75, *p* < 0.028), having very low monthly family income (AOR = 0.22, 95 % CI: 0.06–0.77, *p* < 0.019) and self - purchasing of medications (AOR = 0.30, 95 % CI: 0.10–0.93, *p* < 0.036) were significantly associated with non-adherence.

**Conclusions:**

The study has identified the adherence level to the prescribed ocular hypotensive agents to be sub-optimal and is influenced by different factors among glaucoma patients of the public tertiary center. We recommend glaucoma care providers to pay due attention on the importance of adherence.

## Background

Glaucoma is a progressive optic neuropathy which is caused by the death of the retinal ganglion cells and their fibers [1, 2]. It is a public health problem in developing countries, chiefly in sub-Saharan Africa, which is further compounded by poor awareness and low knowledge of glaucoma patients towards the disease [3, 4].

Adherence is the extent to which a patient’s behavior in taking medication corresponds with agreed recommendations from the provider [5]. Glaucoma medications are used over a long period or life-long that require long-term adherence to achieve the maximum intraocular pressure (IOP) lowering effect and prevent further progression of the disease [1, 6]. The major determinant for success in medical therapy of glaucoma is, therefore, the adherence of patients to their medications [7, 8].

Studies have documented that non-adherence is a significant problem among glaucoma patients which is further influenced by the asymptomatic nature of the disease [9, 10]. Reported adherence varies widely in different glaucoma studies [10] but a systematic review of 31 studies reported that 25 % of patients were found to be non-adherent [11]. Non-adherence potentially results in treatment failure, which is manifested as persistent elevation of IOP which, in turn, leads to progressive optic nerve damage and deterioration of the visual field [12–14]. Non-adherence is also associated with unnecessary prescription of drugs which add an extra burden on the health economy [15].

Glaucoma medication adherence could be measured through self-report, pharmacy refill reports, electronic monitoring and direct observation. To be clinically relevant, an ‘acceptable’ adherence level should be determined by its impact on clinical outcome [10]. Such evidence is lacking for ocular hypotensive agents due to the requirement for long-term follow-up and known inaccuracies in determining IOP control, visual field defects or optic nerve damage [16]. In the absence of any pre-existing gold standard measure, adherence to ocular hypotensive agents was measured using the Morisky Medication Adherence Scale – 8 (MMAS – 8) in this study [17]. The main purpose of this study was, therefore, to assess the level of adherence to ocular hypotensive agents and its determinant factors among glaucoma patients at a tertiary public referral hospital.

## Methods

A hospital-based cross-sectional study was conducted at the glaucoma clinic of Menelik II Referral Hospital, Ethiopia from June 1, 2015 to July 31, 2015. At the clinic, glaucoma diagnosis was made based on the presence of elevated IOP (>20 mmHg), gonioscopy findings, characteristic optic nerve head damage (vertical cup-disc ratio greater than 0.4; diffuse or focal thinning or notching of the neuroretinal rim; or presence of asymmetry of the vertical cup-disc ratio of 0.2 between eyes) and/or visual field defect. The source population for this study was all glaucoma patients who received services at the glaucoma clinic of the hospital and the study population was all glaucoma patients who obtained services during the study period at the clinic.

### Sample size determination and sampling technique

The sample size was calculated using a formula used to estimate the sample size for a single population [18]. Considering 1.96 for the standard normal variable with 5 % level of significance (α - value), 95 % confidence interval, 5 % margin of error and 10 % contingency, the sample size was calculated to be 359 from the 2120 study population. A systematic random sampling technique was employed to select the samples from the study population. The sampling interval was calculated to be 2120/359 = 6. A starting point was chosen randomly from numbers 1 to 6 and hence eligible individuals were chosen every sixth client at regular interval from the sampling frame.

### Recruitment of research participants

Glaucoma patients enter the clinic’s triage and make a queue in the waiting area. The ophthalmic nurses measure both visual acuity and IOP and register these findings on the patient’s chart prior to getting services from physicians. While the patients were waiting at a waiting area during their appointment day, patients were screened for eligibility based on the inclusion and exclusion criteria. The study participants, who were selected from the sampling frame, were briefed about the purpose of the study and then requested for willingness for an interview at a nearby separate room.

### Inclusion and exclusion criteria

Patients who were 18 years old and above, with the diagnosis of glaucoma or ocular hypertension, were on ocular hypotensive agents for one or both eyes for at least 6 months, had regular follow-up and had not undergone either laser or glaucoma surgery in the previous 3 months were enrolled in the study. Glaucoma patients with post-operative follow-up, on systemic glaucoma drugs only, on anti-inflammatory or anti-infective eye drops only, who were not willing to give informed written consent and those enrolled in the pretest were excluded from the study.

### Data collection and analysis

Data were collected by three trained ophthalmic nurses through a face-to-face interview to collect socio-demographic characteristics (age, sex, educational level, residence, and occupation), medication-related characteristics (number, type, and side effects of medications) and adherence level. Medical charts of the patients were reviewed to abstract the type and severity of glaucoma, and visual acuity. Adherence to ocular hypotensive agents was measured using MMAS–8 which is a medication-taking behavior scale. MMAS–8 is the latest version of the scale and has a good internal consistency (Cronbach’s α = 0.83) [17]. This scale has been used for a wide variety of chronic medical conditions [19]. The study participant was deemed to be adherent when the MMAS – 8 score was < 2 and non-adherent when the MMAS – 8 score was ≥ 2 [20].

The questionnaire was translated to Amharic, a national language, and then translated back to English. To maximize quality of the data, the tool was pre-tested in 5 % of the study subjects (18 patients). The filled-in forms were checked for completeness of data and cleaned prior to data entry. Data were entered using Epi Info™ version 3.5.3. Data analysis was carried out using Statistical Package for Social Sciences (SPSS®Statistics) program version 21 (Chicago, IL, U.S.A.). Descriptive statistics such as frequency, percentage, mean and standard deviation were also employed to summarize patient’s characteristics.

Univariate binary logistic regression analysis was performed to assess the association of the variables to adherence. From the result of the univariate analysis, variables with *p* < 0.2 were selected for multivariate binary logistic regression analysis which was used to assess factors affecting adherence and to estimate the odds ratios (OR), 95 % confidence intervals (CI) and *p* - values. The association was declared statistically significant at *p* < 0.05.

## Results

The socio-demographic and clinical characteristics of the study participants are summarized in Table 1. Among 359 eligible patients, about half of the patients (*n* = 181, 50.4 %) were in the age group of 61–80 years old (mean: 60.91, SD ± 12.34 years; range: 18 to 88 years). Large number of the patients were males (*n* = 247, 69.0 %). Concerning the educational level, 229 (63.9 %) patients had a lower education (elementary school and/or below). Majority of the study subjects were residing in urban areas (*n* = 322, 89.7 %) and about one-third (*n* = 115, 32.0 %) of the patients were retired (Table 1).

**Table 1:** Socio-demographic and clinical characteristics of study patients attending the glaucoma clinic, Menelik II Referral Hospital

Variables	Frequency	Percent
Age		
18–40	22	6.1
41–60	149	41.5
61–80	181	50.4
≥ 81	7	1.9
Sex (*n* = 358)		
Male	247	69.0
Female	111	31.0
Educational level (*n* = 358)		
Illiterate or non-formal education	109	30.4
Elementary school (grade 1–8)	120	33.5
High School (grade 9–12)	73	20.4
Diploma and/or above	56	15.6
Place of residence (*n* = 358)		
Rural	36	10.1
Urban	322	89.9
Occupation		
Housewife	29	8.1
Farmer	23	6.4
Retired	115	32.0
Employee (paid work)	79	22.0
Self-employed (merchant)	34	9.5
Other(s) (student, unemployed, prisoner)	79	22.0
Types of glaucoma (*n* = 341)		
Pseudoexfoliative glaucoma	138	40.5
Primary open angle glaucoma	93	27.3
Primary angle closure glaucoma	48	14.1
Other(s) (normal tension glaucoma, secondary glaucoma, ocular hypertension, juvenile glaucoma)	62	18.2
Severity of glaucoma (*n* = 270)		
Early glaucoma	32	11.9
Moderate glaucoma	173	64.1
Advanced glaucoma	65	24.1

According to the medical records of the patients, the most prevalent type of glaucoma diagnosis was pseudoexfoliative glaucoma which accounted for 40.5 % (*n* = 138) followed by primary open angle glaucoma (*n* = 93, 27.3 %). The stage of glaucoma, based on the Canadian glaucoma strategy classification [21], was recorded as early, moderate and advanced in 32 (11.9 %), 173 (64.1 %) and 65 (24.1 %) patients respectively (Table 1).

**Fig. 1:**
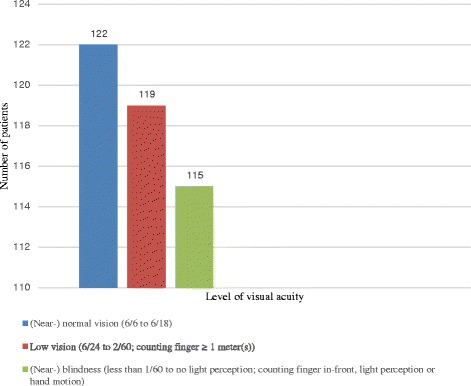
Profile of visual acuity among patients attending the glaucoma clinic, Menelik II Referral Hospital

**Fig. 2:**
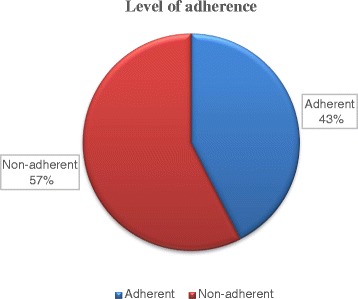
Level of adherence among patients attending the glaucoma clinic, Menelik II Referral Hospital

Almost all (*n* = 350, 97.5 %) of the patients were using one and/or two medications as depicted in Table 2. The combination of eye drops accounted for about half (*n* = 185, 51.5 %) of the prescribed medications followed by timolol as a monotherapy (*n* = 158, 44.0 %). Regarding eye drop administration, 123 (32.8 %) patients admitted that they were waiting more than five minutes to administer the second or consecutive drop. Above half (*n* = 200, 56.0 %,) of the patients also reported that they did not experience side effects (such as redness, itching, burning and blurring of vision) immediately after starting the medications (Table 2). According to the international council of ophthalmology’s classification of visual acuity [22], about one-third of the patients had (near-) normal vision (*n* = 122, 34.3 %), low vision (*n* = 130, 36.6 %) and (near-) blindness (*n* = 115, 32.3 %) on the better eye (Fig. 1). Regarding the level of adherence, assessment of patients’ response to the MMAS-8 revealed that 42.6 % (*n* = 153) of the study patients were adherent to their glaucoma medications (Fig. 2 and Table 3).

**Table 2:** Medication-related factors of glaucoma patients attending Menelik II Referral Hospital

Variables	Frequency	Percent
Number of medications		
One	174	48.7
Two or more	185	51.5
Type of glaucoma medications		
Timolol	158	44.0
Latanoprost	1	0.3
Pilocarpine	2	0.6
Other(s) (dorzolamide, dorzolamide + timolol, brimonidine, betaxolol)	13	3.6
Two or more combination of eye drops	185	51.5
Time elapsed to administer the second or consecutive drop (*n* = 183)		
Immediately	11	6.0
1–5 min	49	26.8
5–10 min	54	29.5
> 10 min	69	37.7
Immediate experience of side effects from the medications (*n* = 357)		
Yes	157	44.0
No	200	56.0

**Table 3:** Glaucoma patients’ response to the eight-item Morisky instrument, glaucoma clinic of Menelik II Referral Hospital

Morisky Medication Adherence Scale (MMAS-8)	Frequency (%)	
	Response	
	Yes	No
Do you sometimes forget to take your eye drop(s)?	209 (58.2)	150 (41.8)
People sometimes miss taking their eye drop(s) for reasons other than forgetting. Thinking over the past 2 weeks, were there any days when you did not apply your eye drop(s)?	116 (32.3)	243 (67.7)
Have you ever cut back or stopped taking your eye drop(s) without telling your doctor because you felt worse when you took it?	19 (5.3)	340 (94.7)
When you travel or leave home, do you sometimes forget to bring along your eye drop(s)?	48 (13.4)	311 (86.6)
Did you take all your eye drop(s) yesterday?	323 (90.0)	36 (10.0)
When you feel like your eye pressure is under control, do you sometimes stop taking your eye drop(s)?	31 (8.6)	328 (91.4)
Applying eye drop(s) every day is a real inconvenience for some people. Do you ever feel hassled about sticking to your treatment plan?	84 (23.4)	275 (76.6)
How often do you have difficulty remembering to apply all your eye drop(s)?		
Never/rarely	140 (39.0)	
Once in a while	137 (38.2)	
Sometimes	56 (15.6)	
Usually	24 (6.7)	
Always	2 (0.6)	
Cut off	Frequency (%)	
< 2	153 (42.6)	
≥ 2	206 (57.4)	

The results of logistic regression analysis for factors associated with medication adherence are summarized in Table 4. Variables with *p* - value less than 0.2 (marital status, educational level, occupation, monthly family income, follow-up, number of medications, type of medications, frequency of eye drop, time elapsed to administer the second drop, side effects, purchasing of the medications, and getting information about eye drop administration from the physicians) were incorporated into the multivariate logistic regression analysis (Table 4).

**Table 4:** Multivariate logistic regression analysis of factors associated with medication adherence among patients attending the glaucoma clinic, Menelik II Referral Hospital

Variables	Adherence		COR (95 % CI)	AOR (95 % CI)
	Non-adherent, *n* (%)	Adherent, *n* (%)		
Educational level				
Illiterate	67 (61.46)	42 (38.54)	1.01 (0.59, 1.72)	2.07 (0.53, 8.13)
Elementary School	74 (61.66)	46 (38.34)	1.00	1.00
High School	38 (52.05)	35 (47.95)	1.48 (0.82, 2.67)	1.87 (0.48, 7.26)
Diploma & above	26 (46.42)	30 (53.57)	1.86 (0.98, 3.53)	4.60 (1.01, 21.03)*
Occupation				
Retired	66 (57.39)	49 (42.61)	1.00	1.00
Farmer	17 (73.9)	6 (26.1)	0.48 (0.16, 1.29)	0.07 (0.01, 0.75)*
House wife	18 (62)	11 (38)	0.82 (0.36, 1.90)	4.40 (0.82, 23.49)
Employee	42 (53.16)	37 (46.84)	1.19 (0.67; 2.11)	2.40 (0.70, 8.21)
Self-employed	16 (47)	18 (53)	1.52 (0.70, 3.27)	6.14 (1.37, 27.50)*
Other(s)	47 (59.5)	32 (40.5)	0.92 (0.51, 1.64)	0.763 (0.15, 3.86)
Monthly family income				
Very low (<445 ETB)	59 (61.45)	37 (38.55)	0.82 (0.49, 1.37)	0.22 (0.06, 0.77)*
Low (446–1200 ETB)	90 (56.6)	69 (43.4)	1.71 (0.78, 3.75)	0.91 (0.18, 4.57)
Average (1201–2500 ETB)	31 (62)	19 (38)	0.80 (0.42, 1.53)	0.31 (0.10, 1.96)
Above average (2501–3500 ETB)	13 (54.16)	11 (45.84)	0.47 (2.61,0.00)	0.62 (0.14, 2.71)
High (>3501 ETB)	13 (43.33)	17 (56.67)	1.00	1.00
Frequency of eye drop				
2 Times per day	3 (75.0)	1 (25.0)	1.85 (0.88, 3.91)	2.89 (1.25, 6.66)*
3 Times per day	35 (67.3)	17 (32.7)	1.27 (0.13, 12.8)	1.77 (0.17, 18.26)
4 Times per day	84 (79.25)	22 (20.75)	1.00	1.00
Getting the medications				
Free of charge	111 (59.7)	75 (40.3)	1.00	1.00
Self-buy	94 (54.65)	78 (45.35)	1.23 (0.81, 1.87)	0.30 (0.10, 0.93)*

*AOR* adjusted odds ratio, *COR* crude odds ratio, *ETB* Ethiopian Birr (one U.S. dollar ≈ 21.05 ETB during the period of data collection)

*Statistically Significant at *p* < 0.05

In this study, educational level, occupation, monthly family income, the frequency of eye drops and financial source to obtain the medications were found to be potential predictors of adherence to ocular hypotensive agents. Accordingly, the odds of being adherent for patients with higher educational level (diploma and/or above) were nearly five-fold (AOR = 4.60, 95 % CI: 1.01–21.03, *p* < 0.049) more compared to patients with lower educational status (Grade 1–8).

The odds of being adherent for farmer patients were 93 % (AOR = 0.07, 95 % CI: 0.01–0.75, *p* < 0.028) less compared to patients who were retired. In contrary to this, patients who were self-employed were approximately six-fold (AOR = 6.14, 95 % CI: 1.37–27.50, *p* < 0.018) more odds of being adherent compared to those participants who were retired. The odds of being adherent for patients with very low monthly family income were 78 % (AOR = 0.22, 95 % CI: 0.06–0.77, *p* < 0.019) less compared to patients with high monthly family income. It was also found that the odds of being adherent for patients who were taking fewer daily doses (two times per day) were approximately three-fold (AOR = 2.89, 95 % CI: 1.25–6.66, *p* < 0.013) more compared to those who were taking more frequent eye drops (four times per day). The odds of being adherent for patients who bought the medications by themselves were about 70 % (AOR = 0.30, 95 % CI: 0.10–0.93, *p* < 0.036) less compared to patients who obtained the medications free of charge.

## Discussions

The study has assessed adherence to ocular hypotensive agents among glaucoma patients in Menelik II Referral Hospital. One hundred fifty-three (42.6 %) patients were found to be adherent which was similar to other studies that reported 40.0–45.0 % in USA [23–25]. This similarity might be evident owing to the use of a questionnaire for interviewing the patients. On the other hand, the level of adherence was found to be lower than 56.0 % in Greece [12], 72.1 % in Canada [26] and 72.7 % in Dutch [27] but higher than 30.7 % in England [28]. The wide variation might be partly attributable to inconsistency in the definition of non-adherence, subjectivity and heterogeneity in the assessment methods as well as differences in patient groups [29].

During multivariate logistic regression analysis, patients with higher educational level were more likely to be adherent compared to patients with lower educational level. This finding was similar to studies done in Canada [26], USA [30, 31] and Germany [32]. Patients who are more literate might have an updated information on the disease and its progression, and have a better understanding of the importance of adherence to the medications. On the other point, self-employed patients were more likely to be adherent compared to retired patients as the former might afford the medications easily. Moreover, retired patients are usually older than self-employed patients and might have greater cognitive and physical impairment associated with aging.

In contrary to the above, farmer patients were less likely to be adherent compared to retired patients. This could be illustrated as the majority of the farmers in developing countries like Ethiopia are illiterate with limited knowledge of the disease and have lower economic status.

Patients who applied eye drops less frequently were more likely to be adherent compared to patients who applied eye drops more frequently which corresponded with a previous study [12]. This is evident by the fact that increased daily frequency of eye drop is associated with increased complexity of the regimen. However, more emphasis should be given to the clinical importance of frequency of administration and available dosage preparations.

Patients with low monthly income were less likely to be adherent compared to patients with high monthly income. This finding was related to a previous study that revealed unaffordability considerably affects adherence [33]. Besides this, patients who purchased ocular hypotensive drops by themselves were less likely to be adherent compared to patients who obtained their medications free of charge. The plausible reason might also be related to an affordability issue of the medications.

In this study, age and sex of the participants were not significantly associated with the medication adherence during the multivariate logistic regression analysis. The absence of a relationship between adherence and most of the socio-demographic factors was supported by the previous findings [12, 23, 27, 29, 32, 34]. The absence of this association might be related to the characteristics of patients. The patients in the present study had a long history of glaucoma (for an average of 5.6 years) and a long duration of taking medication (mean duration of taking hypotensive agents was 5.35 years). Therefore, demographic factors might have less influence on the adherence behavior of the study participants.

Multivariate analysis of this study also indicated that medication adherence was not significantly associated with the type and severity of glaucoma, side effects (even though side effects were reported most commonly amongst those who were non-adherent), duration of glaucoma diagnosis, obtaining information about drug administration, intraocular pressure, and visual acuity. These results were comparable with different studies [12, 27, 33–35]. It remains unclear whether these clinical parameters might influence the adherence behavior of glaucoma patients in general.

The study had certain limitations. The cross-sectional nature of the study did not allow a follow-up, which could have provided a better design for identifying the factors associated with adherence. The results were also relied on patients’ response. It is known that patients tend to overestimate their ability to adhere to their therapy which necessities further objective assessment tools such as electronic medication monitoring system or biological assays in the future.

## Conclusion

The study has identified the adherence level to the prescribed ocular hypotensive agents to be sub-optimal according to the Morisky Medication Adherence Scale-8, and influenced by different factors among glaucoma patients of the public tertiary center. We recommend glaucoma care providers to pay due attention to the importance of adherence and influencing factors to the prescribed medications.

## Abbreviations

AOR, adjusted odds ratio; CI, confidence interval; COR, crude odds ratio; IOP, intraocular pressure; MMAS – 8, Morisky Medication Adherence Scale – 8; SD, standard deviation
